# Systematic Optimization of Monomer System and Bimodal Particle Size Distribution for Alumina Slurries in DLP Additive Manufacturing

**DOI:** 10.3390/ma19173703

**Published:** 2026-08-31

**Authors:** Jin Hyun Kim, Jung Hoon Choi, Jin Ho Kim, Kyu Sung Han, Ung Soo Kim

**Affiliations:** Korea Institute of Ceramic Engineering and Technology, Icheon 17303, Republic of Korea; michael41300@gmail.com (J.H.K.); eunu@kicet.re.kr (J.H.C.); jino.kim@kicet.re.kr (J.H.K.); kh389@kicet.re.kr (K.S.H.)

**Keywords:** photopolymerization, rheological behavior, packing density, dimensional accuracy, densification

## Abstract

Digital light processing (DLP)-based additive manufacturing of ceramics requires photocurable slurries with high solid loading, low viscosity, and excellent photocuring behavior. In this study, the effects of monomer composition and optimized bimodal alumina powder formulations on the rheological behavior, photocuring characteristics, printability, and sintering behavior of high-solid-loading alumina slurries were investigated. Five commercial photocurable monomers with different functionalities were evaluated to optimize the resin formulation. Among them, a binary monomer system consisting of 2-hydroxyethyl acrylate (2-HEA) and 1,6-hexanediol diacrylate (1,6-HDDA) at a weight ratio of 6:4 exhibited a favorable combination of low viscosity and photopolymerization behavior under the investigated conditions. Using this optimized resin, 50 vol.% alumina slurries containing two optimized bimodal powder formulations (2 μm/100 nm and 4 μm/400 nm) were prepared. The 4 μm/400 nm formulation showed higher double-bond conversion during UV curing, whereas the finer 2 μm/100 nm formulation produced higher relative density after sintering because of its superior packing efficiency. The optimized binary monomer system also suppressed lateral over-curing, resulting in improved dimensional accuracy compared with the single-monomer resin. Among all formulations, the slurry containing the 2 μm/100 nm bimodal powder and the optimized binary monomer system exhibited the best overall performance, including stable printing, minimal delamination, the highest relative density, and uniform sintering shrinkage. These findings provide practical design guidelines for the formulation and processing of high-solid-loading photocurable alumina slurries for DLP additive manufacturing.

## 1. Introduction

Ceramic additive manufacturing using photocurable slurries is a fabrication technology in which ceramic powders are highly dispersed in a photocurable resin and selectively photopolymerized to produce ceramic components with complex geometries [1,2,3,4,5,6]. Representative vat photopolymerization processes include stereolithography (SLA), digital light processing (DLP), two-photon polymerization (2PP), and UV-assisted direct ink writing (DIW). Among these techniques, DLP has become the most widely adopted because of its high printing speed, excellent dimensional accuracy, and superior geometric freedom. It has been successfully applied to a wide range of ceramic materials, including Al_2_O_3_, ZrO_2_, and Si_3_N_4_, for the fabrication of high-performance ceramic components used in semiconductor and energy applications, as well as fillers in dental resins. However, in DLP-based ceramic processing, the slurry viscosity, dispersion stability, photopolymerization behavior, and sintering shrinkage are closely interrelated, and their optimization is critical for achieving high densification and dimensional accuracy in the final ceramic components [7,8,9,10].

The composition of the photocurable resin is one of the most important factors governing both the rheological properties and photopolymerization behavior of ceramic slurries [11,12,13,14,15]. Acrylate-based monomers such as 1,6-hexanediol diacrylate (1,6-HDDA), polyethylene glycol diacrylate (PEGDA), and trimethylolpropane triacrylate (TMPTA) are commonly employed in DLP systems. Monofunctional monomers generally exhibit low viscosity, thereby improving slurry flowability, whereas multifunctional monomers form highly crosslinked polymer networks that enhance the mechanical strength of green bodies. However, the increased crosslink density is often accompanied by greater polymerization shrinkage and higher internal stress. Furthermore, photopolymerization behavior is influenced not only by the number of functional groups but also by molecular structure and chain mobility. Consequently, binary monomer systems consisting of monomers with complementary characteristics have recently attracted considerable attention as an effective strategy for simultaneously achieving low viscosity and excellent photocuring performance [16,17,18,19].

The particle size distribution of ceramic powders is another key design parameter in DLP [20,21,22,23,24]. Bimodal particle systems composed of coarse and fine particles generally provide higher packing density and improved sinterability. Nevertheless, the increased specific surface area associated with fine particles also affects dispersant demand, light scattering, and photocuring behavior. Therefore, both the monomer system and particle size distribution play decisive roles in determining the rheological behavior of ceramic slurries, photocuring efficiency, printing resolution, and the final sintering characteristics. Despite their importance, previous studies have primarily focused on either the photocurable resin or the powder formulation independently, whereas studies evaluating both factors throughout the DLP process remain limited.

In this study, the viscosities and photopolymerization behaviors of five monomers with different functionalities and molecular structures, together with various binary monomer systems, were systematically evaluated to identify the optimum resin composition. Subsequently, 50 vol.% photocurable Al_2_O_3_ slurries incorporating different bimodal particle size distributions were prepared, and their rheological properties, photocuring behavior, and DLP printability were investigated. Furthermore, the debinding and sintering behaviors, relative density, and directional shrinkage of printed honeycomb structures were compared to evaluate the influence of the selected resin and optimized bimodal powder formulations on DLP-based alumina additive manufacturing. Based on these findings, practical design guidelines for high-solid-loading, low-viscosity photocurable alumina slurries compatible with commercial DLP printing systems are proposed.

## 2. Experimental Procedure

### 2.1. Materials

Five commercially available photocurable monomers with different functionalities (f) were used in this study: 2-hydroxyethyl acrylate (2-HEA, *f* = 1, 116.1 g mol^−1^), 2-hydroxyethyl methacrylate (2-HEMA, *f* = 1, 130.1 g mol^−1^), bisphenol A ethoxylate diacrylate (BPAEDA, *f* = 2, 424.2 g mol^−1^), 1,6-hexanediol diacrylate (1,6-HDDA, *f* = 2, 226.3 g mol^−1^), and trimethylolpropane triacrylate (TMPTA, *f* = 3, 296.2 g mol^−1^). All monomers were purchased from Sigma-Aldrich (St. Louis, MO, USA). Phenylbis(2,4,6-trimethylbenzoyl) phosphine oxide (BAPO, Sigma-Aldrich, St. Louis, MO, USA) was used as the photoinitiator, and DISPERBYK-111 (BYK-Chemie, Wesel, Germany) was employed as the dispersant.

Four alumina powders with different average particle sizes were used as ceramic raw materials: AM-21 (4 μm, Sumitomo Chemical, Tokyo, Japan), AES-23 (2 μm, Sumitomo Chemical, Tokyo, Japan), AES-11 (400 nm, Sumitomo Chemical, Tokyo, Japan), and 26N-0811UPA (100 nm, Inframat Advanced Materials, Amherst, MA, USA).

### 2.2. Optimization of Photocurable Resin Composition

The viscosities of single monomers and various binary monomer systems were compared to identify the optimum resin composition. Binary monomer systems were prepared by mixing 2-HEA with 1,6-HDDA, BPAEDA, or TMPTA at different weight ratios. To ensure homogeneous mixing, the monomers were stirred at 180 rpm for 24 h at room temperature using a magnetic stirrer.

The viscosities of the monomers were measured using a rotational rheometer (MCR 302, Anton Paar, Graz, Austria) equipped with a 50 mm parallel-plate geometry at 25 °C. The viscosity was measured over a shear rate range of 0.1–100 s^−1^. For photocuring characterization, 1 wt.% BAPO was added to each single or binary monomer system, followed by magnetic stirring at 100 rpm for 24 h. The photopolymerization behavior was evaluated by measuring the polymerization enthalpy (ΔH_p_) under UV irradiation for 1–10 s using a Photo-DSC instrument (DSC 204 F1 Phoenix, NETZSCH, Selb, Germany). The heat-flow curves obtained at a UV intensity of 1 mW cm^−2^ were analyzed using NETZSCH Proteus software version 7.0. Baseline correction was performed using a linear baseline connecting the onset and end of the photopolymerization exotherm, and ΔH_p_ was determined by integrating the baseline-corrected exothermic peak. This DSC-based approach follows the methodology reported by Stanko and Stommel [25], in which reaction progress is evaluated from the integrated reaction enthalpy. Because the investigated monomers differ in molecular weight and functionality, the ΔH_p_ values expressed in J/g were not normalized to the molar concentration of reactive C=C bonds. Therefore, these values were used to compare the photopolymerization behavior of the monomer systems under identical irradiation conditions rather than as a measure of intrinsic monomer reactivity. All Photo-DSC measurements were performed three times, and average values were reported.

### 2.3. Preparation of Photocurable Alumina Slurries

Two bimodal Al_2_O_3_ powder mixtures with different particle size distributions were prepared. Based on our preliminary evaluation of the packing characteristics as a function of the coarse-to-fine particle ratio, the mixing ratio providing the highest packing density was selected for each bimodal powder system. Accordingly, the 2 μm and 100 nm powders were blended at a weight ratio of 7:3, whereas the 4 μm and 400 nm powders were mixed at a weight ratio of 6:4.

To promote preferential adsorption of the dispersant onto the particle surfaces, the alumina powders were mixed with ethanol at a weight ratio of 1:2. DISPERBYK-111 was then added at concentrations of 2, 4, 6, and 8 mg/m^2^ based on the specific surface area of the powder mixtures. The suspensions were ball-milled at 160 rpm for 24 h and subsequently dried at 60 °C for 4 h to completely remove the ethanol.

The dried alumina powders (50 vol.%) were dispersed in a binary monomer resin consisting of 2-HEA and 1,6-HDDA (6:4 by weight). Ball milling was then performed at 5 rpm for 24 h to prepare the alumina slurries. The rheological properties of the slurries were evaluated using the rheometer described above, and the dispersant concentration yielding the lowest viscosity was selected as the optimum dispersion condition.

After determining the optimum dispersant concentration, 1 wt.% BAPO was added to each slurry, followed by stirring at 100 rpm for 24 h to prepare the photocurable alumina slurries. According to the powder combination and resin composition, the slurries were designated as 4B (4 μm + 400 nm Al_2_O_3_ with binary monomer), 4S (4 μm + 400 nm Al_2_O_3_ with single monomer), 2B (2 μm + 100 nm Al_2_O_3_ with binary monomer), and 2S (2 μm + 100 nm Al_2_O_3_ with single monomer).

The photocuring behavior of each slurry was analyzed using Photo-DSC. The degree of conversion was calculated from the measured total polymerization enthalpy [25].

### 2.4. DLP Printing and Heat Treatment

The printability of the four photocurable alumina slurries was evaluated by fabricating honeycomb structures using a commercial DLP-based vat photopolymerization 3D printer (IM-96, Carima, Seoul, Republic of Korea). The layer thickness was fixed at 50 μm, and the initial exposure time was set to 40 s. The normal exposure time was varied from 0.5 to 1.5 s to evaluate printability, while the UV wavelength and light intensity were maintained at 405 nm and 6 mW cm^−2^, respectively. Based on the printing results, a normal layer exposure time of 0.8 s was selected for the specimens subsequently used for TG-DTA analysis, debinding, and sintering. Eight initial layers were printed, followed by 200 additional layers to fabricate the final specimens. Three independently printed specimens were evaluated for each formulation, and the curing width is reported as the mean ± standard deviation (*n* = 3).

The thermal decomposition behavior of the printed honeycomb structures was investigated by simultaneous thermogravimetric and differential thermal analysis (TG-DTA; STA7200RV, Hitachi, Tokyo, Japan) to optimize the debinding schedule. Measurements were performed using alumina crucibles over the temperature range of 25–800 °C at a heating rate of 5 °C/min.

Based on the TG-DTA results, debinding was carried out by heating to 550 °C at a rate of 1 °C/min. Subsequently, the specimens were heated to 1600 °C at 3 °C/min and sintered for 5 h. The relative density of the sintered specimens was determined by the Archimedes method. The linear sintering shrinkage of the honeycomb structures was evaluated by measuring the dimensions along the X-, Y-, and Z-directions before and after sintering using a digital Vernier caliper. Each dimension was measured three times, and the average value was used for calculating the shrinkage.

## 3. Results and Discussion

### 3.1. Optimization of the Photo-Curable Resin Composition

The viscosities of the single monomers were measured as 8 mPa·s for 2-HEA, 8 mPa·s for 2-HEMA, 9 mPa·s for 1,6-HDDA, 2295 mPa·s for BPAEDA, and 123 mPa·s for TMPTA at a shear rate of 1 s^−1^. In general, the monomer viscosity increased with increasing molecular weight and structural complexity. Accordingly, 2-HEA, 2-HEMA, and 1,6-HDDA, which exhibited relatively low viscosities, were considered suitable for preparing ceramic slurries with high solid loadings.

**Figure 1:**
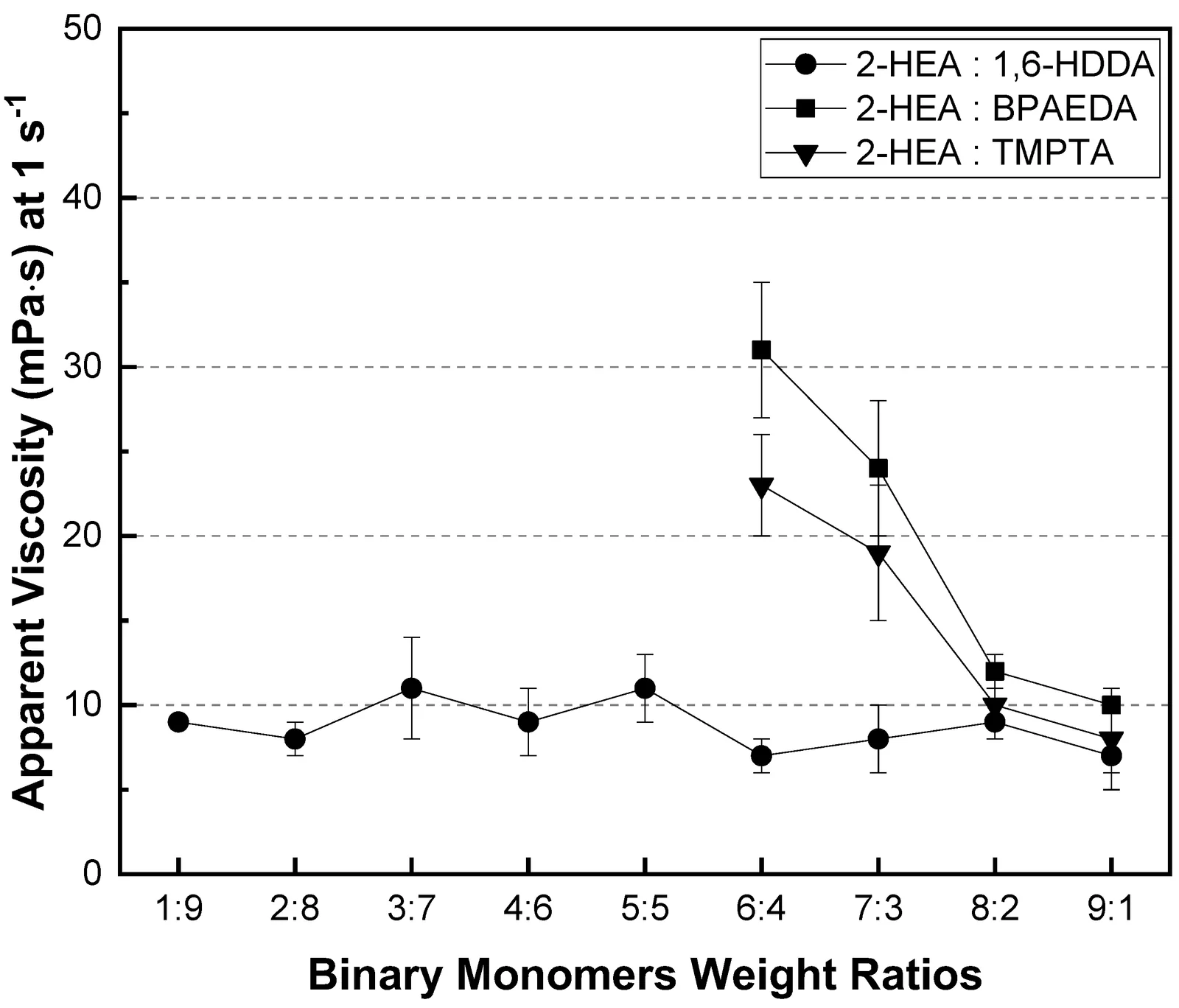
Viscosity changes in binary monomer systems prepared by blending 2-HEA with the difunctional monomers 1,6-HDDA and BPAEDA or with the trifunctional monomer TMPTA.

Figure 1 also shows the viscosity changes in binary monomer systems prepared by blending 2-HEA with the difunctional monomers 1,6-HDDA and BPAEDA or with the trifunctional monomer TMPTA. The 2-HEA/1,6-HDDA system maintained a low viscosity of less than 11 mPa·s over the entire composition range. In particular, the viscosity remained below 10 mPa·s at 2-HEA:1,6-HDDA weight ratios of up to 6:4. In contrast, the viscosities of the systems containing BPAEDA or TMPTA increased as the proportion of the multifunctional monomer increased. BPAEDA caused the most pronounced increase in viscosity because of its aromatic structure and intrinsically high viscosity. Therefore, 1,6-HDDA was considered the most suitable multifunctional monomer for photocurable ceramic resins because it provided both low viscosity and multifunctionality.

**Figure 2:**
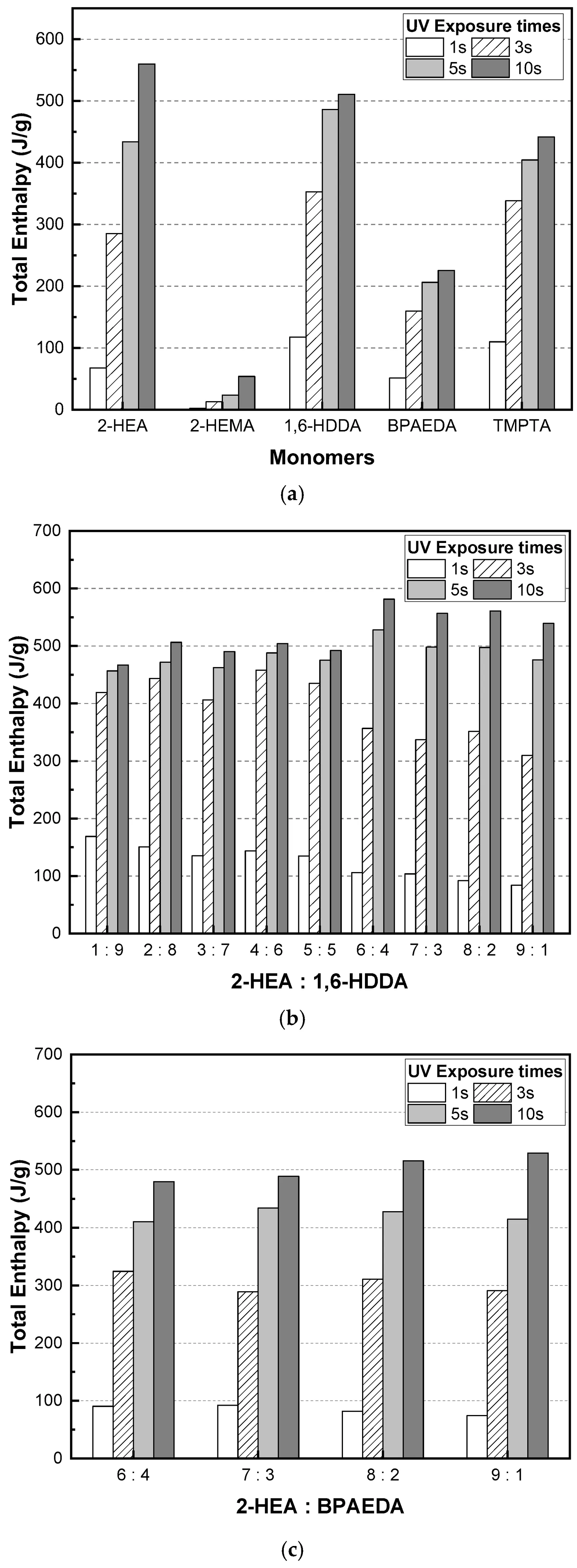

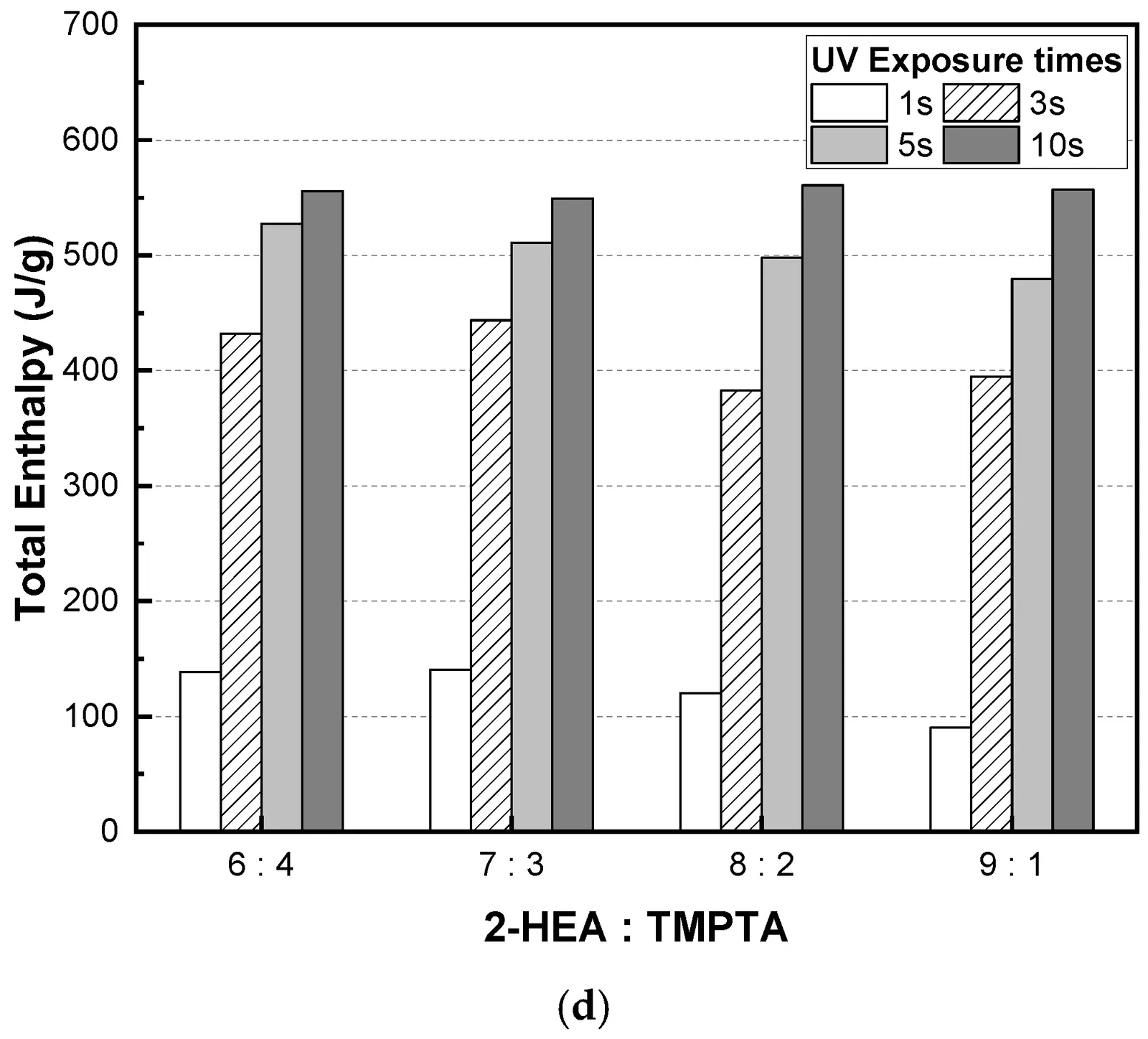
Total enthalpy of single and binary monomers with increasing UV exposure time: (**a**) single monomers; (**b**) 2-HEA/1,6-HDDA binary monomer system; (**c**) 2-HEA/BPAEDA binary monomer system; (**d**) 2-HEA/TMPTA binary monomer system.

Figure 2a shows the photopolymerization behavior of the single monomers containing 1 wt.% BAPO, evaluated from the variation in total polymerization enthalpy as a function of UV irradiation time from 1 to 10 s. For all monomers, the total enthalpy initially increased rapidly with irradiation time and then gradually approached a plateau. After 10 s of UV irradiation, the measured polymerization enthalpy decreased in the order 2-HEA > 1,6-HDDA > TMPTA > BPAEDA ≫ 2-HEMA. It should be noted that these mass-normalized enthalpy values do not represent intrinsic monomer reactivity because the monomers differ in molecular weight and functionality. Nevertheless, the relatively low polymerization enthalpy of 2-HEMA under the present experimental conditions is consistent with the lower photopolymerization tendency of methacrylates associated with steric hindrance by the α-methyl group [26]. For the multifunctional monomers, the molecular structure exerted a greater influence on the photopolymerization behavior than the number of functional groups alone. The linear molecular structure of 1,6-HDDA provided relatively high chain mobility and resulted in a high total polymerization enthalpy. In contrast, BPAEDA exhibited the lowest polymerization enthalpy among the multifunctional monomers under the present experimental conditions, which may be associated with its high viscosity and steric hindrance associated with its aromatic backbone. Although TMPTA formed a polymer network with a high crosslink density, its final total enthalpy was lower than that of 1,6-HDDA because diffusion was restricted by early-stage vitrification. These results agree well with previous studies showing that the photopolymerization of multifunctional acrylates is strongly influenced by molecular structure and diffusion limitations [27,28,29,30].

Figure 2b–d presents the photopolymerization behavior of the binary monomer systems. For all compositions, the total enthalpy increased rapidly during the initial irradiation period of 1–3 s and subsequently approached a plateau. This behavior can be explained by the rapid initiation and propagation of photopolymerization through free radicals during the early stage, followed by restricted diffusion of the monomers and radicals as the crosslinked network developed [31]. The 2-HEA/1,6-HDDA system maintained relatively high polymerization enthalpy over the widest composition range, and the measured enthalpy increased with increasing 2-HEA content. Conversely, an increase in the 1,6-HDDA content reduced the overall extent of polymerization because early crosslinking restricted molecular diffusion. The systems containing BPAEDA exhibited the lowest polymerization enthalpy over the entire composition range. Although the TMPTA-containing systems showed rapid initial photopolymerization, their final total enthalpy was limited by early-stage vitrification.

Overall, the binary monomer system consisting of 2-HEA and 1,6-HDDA at a weight ratio of 6:4 provided a favorable combination of low viscosity and photopolymerization behavior under the investigated conditions. This composition was therefore selected for the subsequent preparation of photocurable alumina slurries.

### 3.2. Rheological and Photocuring Behavior of Alumina Slurries

**Figure 3:**
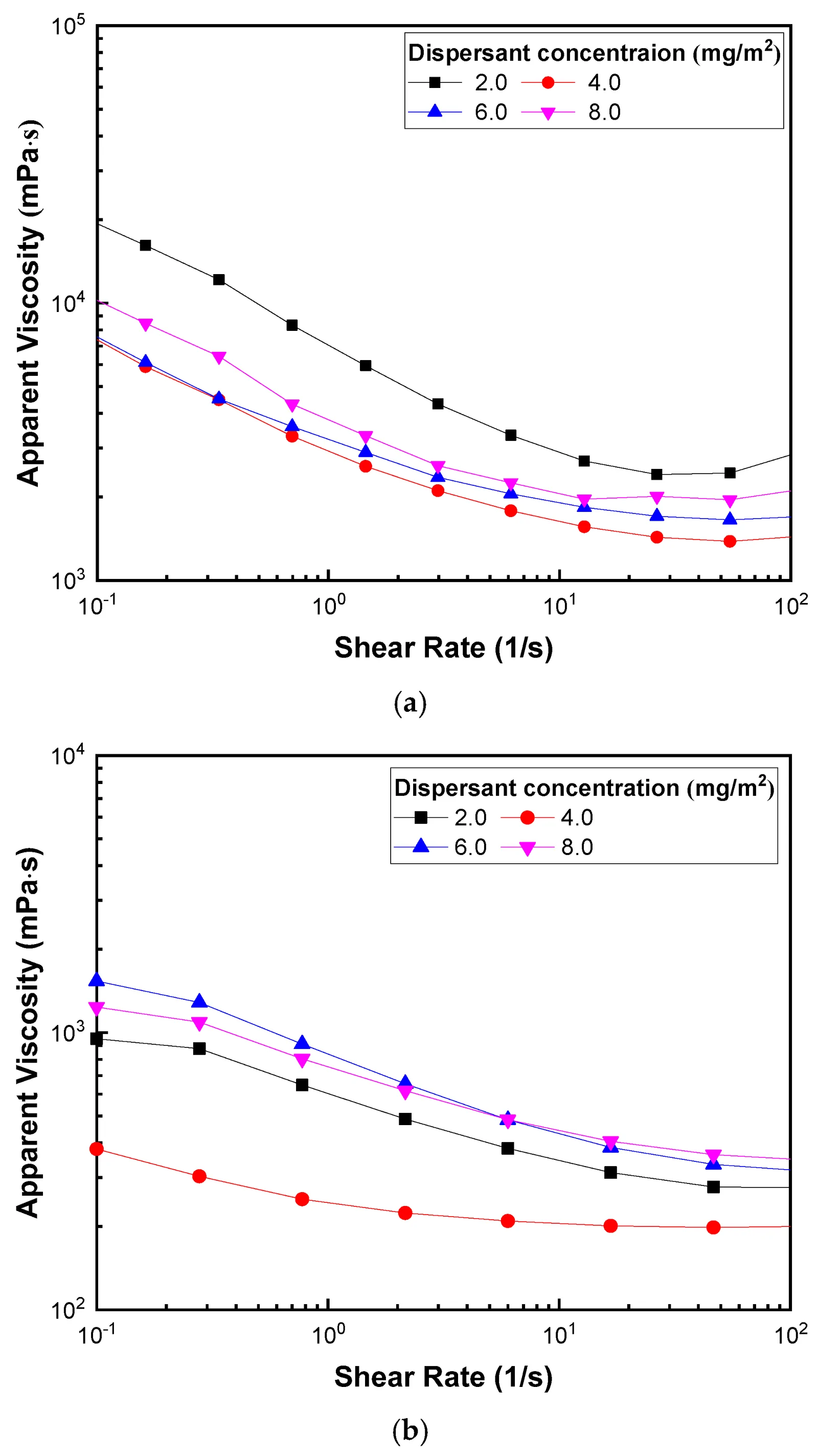
Rheological behavior of 50 vol.% photocurable alumina slurries with varying dispersant contents in a binary monomer resin composed of 2-HEA and 1,6-HDDA (6:4 by weight): (**a**) 2 μm/100 nm alumina system with a weight ratio of 7:3 and (**b**) 4 μm/400 nm alumina system with a weight ratio of 6:4.

Figure 3 shows the viscosity of the 50 vol.% photocurable alumina slurries prepared using BYK-111 as a dispersant as a function of shear rate. All slurries exhibited typical shear thinning behavior, in which the viscosity decreased with increasing shear rate. This behavior was attributed to the adsorption of BYK-111 onto the alumina particle surfaces, which suppressed interparticle agglomeration, and to the gradual breakdown of the particle network under increasing shear, thereby improving slurry flowability [32]. Such shear thinning behavior is advantageous for DLP because it facilitates uniform recoating and surface leveling between consecutive layers.

The 2 μm/100 nm system mixed at a weight ratio of 7:3 (Figure 3a) showed relatively small changes in viscosity with dispersant concentration. In particular, similar flow curves were obtained at dispersant concentrations of 4–6 mg/m^2^, indicating a relatively broad processing window. At 8 mg/m^2^, however, the viscosity increased slightly, presumably because excess dispersant remained in the continuous resin phase. In contrast, the 4 μm/400 nm system mixed at a weight ratio of 6:4 (Figure 3b) was more sensitive to the dispersant concentration and exhibited the lowest viscosity at 4 mg/m^2^. At lower concentrations, insufficient surface coverage by the dispersant promoted particle agglomeration, whereas at higher concentrations, excess dispersant caused the viscosity to increase again. Accordingly, 4 mg/m^2^ was selected as the optimum dispersant concentration for both particle size systems. The lower sensitivity of the 2 μm/100 nm system to the dispersant concentration may be associated with differences in particle arrangement and packing behavior arising from its specific bimodal powder formulation.

**Figure 4:**
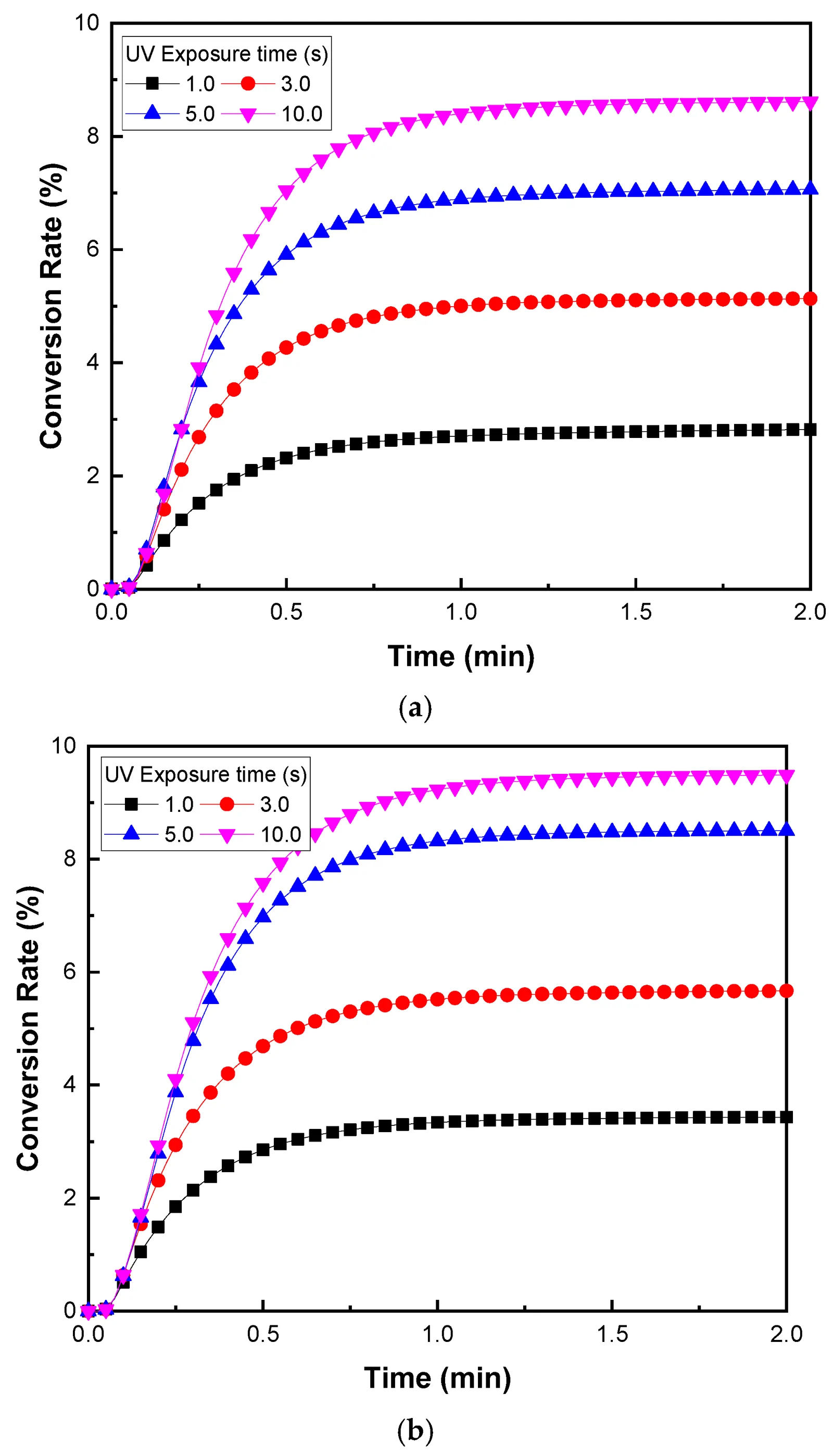
Double-bond conversion of photocurable alumina slurries as a function of UV exposure time: (**a**) slurry containing a 2 μm/100 nm alumina powder mixture (7:3 by weight) and (**b**) slurry containing a 4 μm/400 nm alumina powder mixture (6:4 by weight). Both slurries were prepared using a binary monomer system (2-HEA:1,6-HDDA = 6:4 by weight), 1 wt.% BAPO photoinitiator, and a dispersant content of 4.0 mg/m^2^.

Figure 4 shows the degree of double-bond conversion as a function of UV irradiation time for the slurries prepared at the optimum dispersant concentration of 4 mg/m^2^. For both particle size systems, the conversion increased rapidly with irradiation time and then gradually approached a plateau after approximately 5 s. During the early stage of irradiation, photopolymerization proceeded rapidly because of free radicals generated from BAPO. As the reaction progressed, however, the formation of a crosslinked polymer network reduced the mobility of the monomers and radicals, resulting in a gradual decrease in the polymerization rate [31].

Differences in photopolymerization behavior were observed between the two particle size combinations. The 4 μm/400 nm system (Figure 4b) exhibited a higher degree of double-bond conversion than the 2 μm/100 nm system (Figure 4a) over the entire irradiation period, and the difference became more pronounced during the later stages of the reaction. The higher specific surface area of the 2 μm/100 nm system may have contributed to this behavior by increasing the particle–resin interfacial area and thereby restricting the mobility of the monomers and free radicals. In comparison, the 4 μm/400 nm system had a smaller interfacial area, allowing photopolymerization to proceed more efficiently.

These results demonstrate that the two bimodal powder formulations exhibited different rheological and photopolymerization characteristics. The 2 μm/100 nm system exhibited comparatively stable rheological behavior over a broad range of dispersant concentrations, whereas the 4 μm/400 nm system exhibited a higher degree of double-bond conversion. Therefore, in photocurable ceramic slurries for DLP, the particle size distribution simultaneously governs the rheological behavior and photopolymerization efficiency. Slurry formulations should consequently be designed by balancing these two characteristics.

### 3.3. Printing Performance and Dimensional Accuracy of Photocurable Alumina Slurries

**Figure 5:**
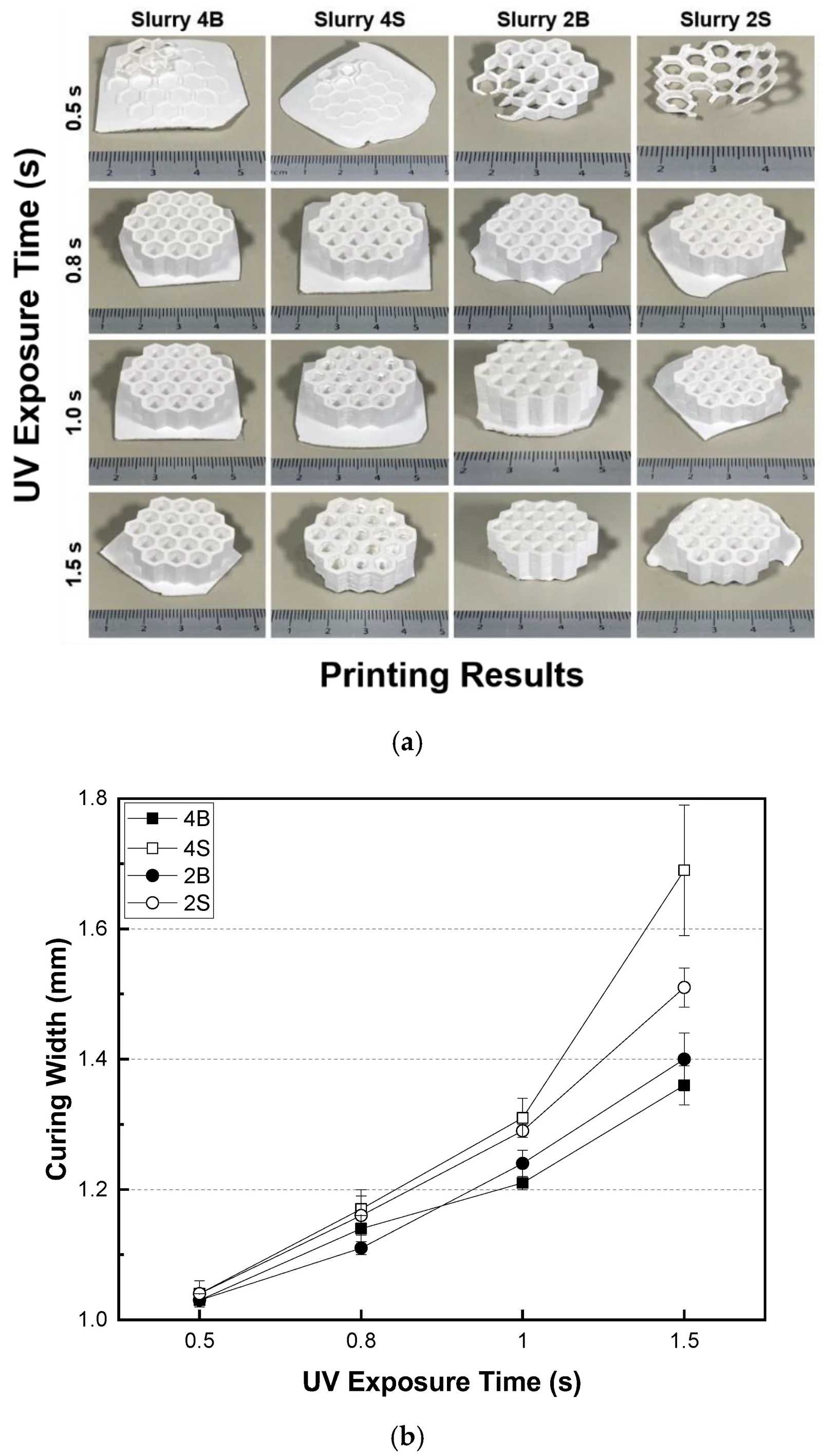
DLP printing behavior of four photocurable alumina slurries under different UV exposure times: (**a**) optical images of the printed honeycomb green bodies and (**b**) measured curing width of the green bodies as a function of UV exposure time. Slurry 4B and 4S were prepared using the 4 μm/400 nm alumina system with binary and single monomer resins, respectively, whereas slurry 2B and 2S were prepared using the 2 μm/100 nm alumina system with binary and single monomer resins, respectively.

The DLP printing behavior and curing width of the photocurable Al_2_O_3_ slurries are shown in Figure 5. At a UV exposure time of 0.5 s, the irradiation energy was insufficient to form well defined honeycomb structures for any of the slurries, and adequate interlayer bonding could not be maintained. In contrast, stable honeycomb structures were successfully fabricated from all slurries at an exposure time of 0.8 s, and this condition was therefore selected for subsequent thermal analysis and sintering experiments. When the exposure time was increased to 1.0 s or longer, the structures remained mechanically stable; however, shape broadening was observed at the edges and outer regions because of lateral over-curing. This phenomenon was attributed to the simultaneous increase in the penetration depth of the incident light and the lateral extent of the photopolymerized region with increasing exposure time [33,34].

Figure 5b shows the variation in curing width with UV exposure time. For all slurries, the curing width gradually increased with exposure time, and the differences among the slurry formulations became more apparent at exposure times of 1.0 s or longer. In particular, the single monomer systems, 4S and 2S, exhibited larger curing widths than the binary monomer systems, 4B and 2B, under all exposure conditions. The curing width of 4S increased to approximately 1.69 mm at an exposure time of 1.5 s, considerably exceeding the designed width of 1.0 mm. In contrast, 4B exhibited the smallest curing width under the same conditions, indicating that excessive lateral curing was effectively suppressed. These results suggest that blending 2-HEA with 1,6-HDDA regulated both the photopolymerization rate and the spatial propagation of the cured region, thereby reducing lateral over-curing compared with the single monomer system.

The effect of the bimodal powder formulations on the curing width was less pronounced than that of the resin composition. Although some differences in curing width were observed between the particle size systems prepared using the same resin composition, no consistent increasing or decreasing trend was identified. This finding indicates that the curing width was not determined solely by particle size but instead resulted from the combined effects of light scattering, light absorption, particle–resin interfacial area, and photopolymerization kinetics. Therefore, within the formulations investigated in this study, the resin composition showed a greater influence on the curing width and dimensional accuracy than the bimodal powder formulation.

Taken together, the results in Figure 5a,b show that the binary monomer slurries, 2B and 4B, provided more stable layer formation and higher dimensional accuracy than the corresponding single monomer slurries. These findings demonstrate that the resin composition had a greater influence on the curing width than the particle size combination and confirm that the 2-HEA/1,6-HDDA binary system is suitable for DLP-based additive manufacturing of alumina ceramics.

### 3.4. Debinding and Sintering Behavior of Printed Alumina Green Bodies

**Figure 6:**
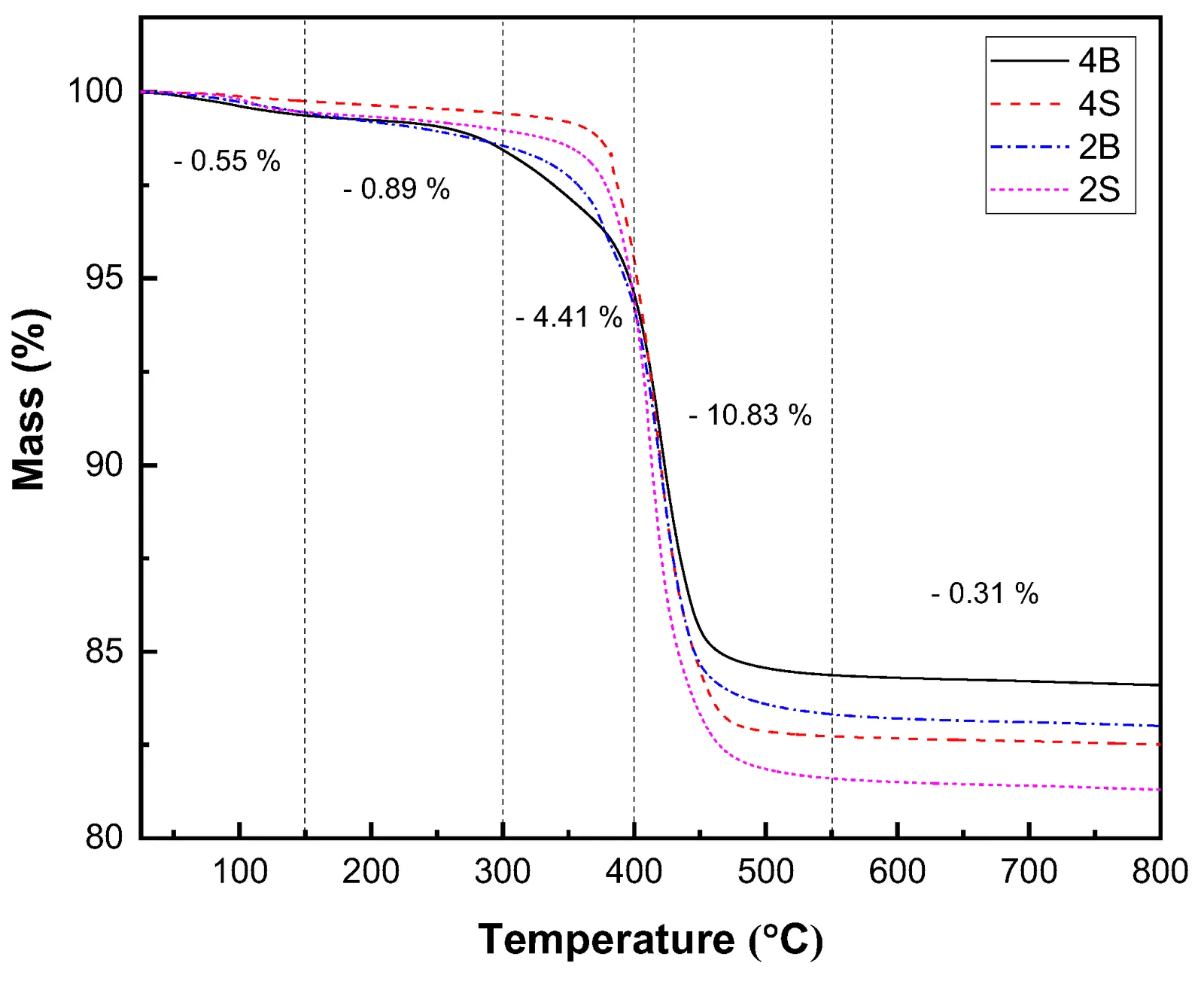
Thermogravimetric (TG) mass−loss curves of the four photocurable alumina slurries during heating.

The thermal decomposition behavior of the printed green bodies is shown in Figure 6. All specimens exhibited similar mass-loss behavior, which could be divided into three temperature regions. Below 150 °C, residual moisture and volatile components were removed. Between 150 and 500 °C, the unreacted monomers and organic binders constituting the photocured polymer network decomposed sequentially, with the largest mass loss occurring between 400 and 500 °C. Above 500 °C, negligible additional mass loss was observed, indicating that most of the organic components had been removed below 500 °C.

Although the overall thermal decomposition behavior was similar for all resin formulations, the single monomer systems, 4S and 2S, exhibited slightly lower residual masses than the binary monomer systems, 4B and 2B. Under the same resin conditions, the slurries containing the 2 μm and 100 nm powders also exhibited slightly lower residual masses than those containing the 4 μm and 400 nm powders. These differences in residual mass may be related not only to differences in the burnout behavior of the monomers but also to variations in binder distribution associated with the particle size distribution. Despite the small differences in the final residual mass, the overall decomposition temperature ranges and the principal mass-loss regions were similar among the specimens. These results suggest that rapid additional gas evolution is unlikely above 500 °C and that stable debinding can be achieved when an appropriately slow heating rate is employed.

**Figure 7:**
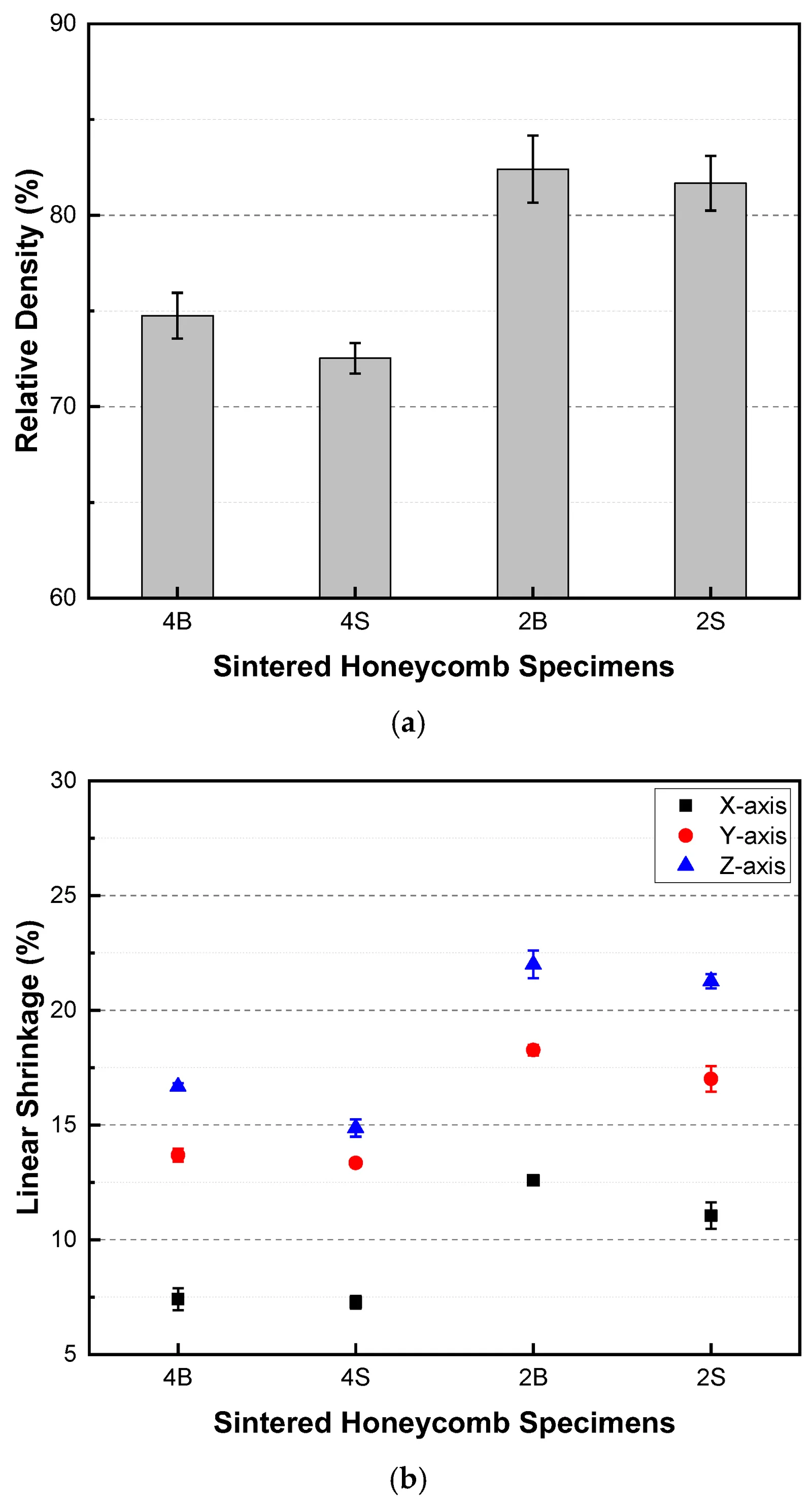
Sintering characteristics of honeycomb specimens fabricated from the four photocurable alumina slurries after sintering at 1600 °C: (**a**) relative density and (**b**) linear shrinkage in the X-, Y-, and Z-directions.

Figure 7a shows the relative densities of the specimens sintered at 1600 °C. Under identical particle size conditions, the binary monomer systems exhibited relative densities approximately 1–2% higher than those of the corresponding single monomer systems. However, within the formulations investigated, the bimodal powder formulation showed a greater influence on the final densification than the resin composition. The specimens prepared using the 2 μm/100 nm bimodal powder system exhibited higher relative densities than those prepared using the 4 μm/400 nm system. The higher specific surface area of the finer particles may have contributed to the greater sintering driving force, while differences in particle packing associated with the respective bimodal formulations may also have influenced densification [35]. In addition, the binary monomer system may have produced more homogeneous green bodies, thereby suppressing defect formation during sintering and promoting slightly greater densification under identical particle size conditions.

Figure 7b shows the linear shrinkage of the sintered specimens along the X-, Y-, and Z-directions. All specimens exhibited shrinkage in the order X < Y < Z, with the largest shrinkage occurring along the build direction, corresponding to the Z-axis. This result reflects the anisotropic sintering behavior associated with the layer-by-layer architecture produced by the DLP process. The 2 μm/100 nm system exhibited both higher relative density and greater shrinkage, which is consistent with its enhanced densification behavior. In contrast, the binary monomer systems showed slightly smaller differences in shrinkage among the three directions under identical particle size conditions. This result suggests that the more homogeneous green body structure produced by the binary resin improved dimensional stability during sintering.

Overall, these results indicate that, within the formulations investigated, the bimodal powder formulation had a greater influence on final densification and sintering shrinkage than the resin system. In particular, the 2-HEA/1,6-HDDA binary monomer system produced comparatively homogeneous green bodies through stable photocuring and debinding behavior and contributed to improved densification and dimensional stability under identical particle size conditions.

## 4. Conclusions

This study investigated the effects of monomer composition and optimized bimodal alumina powder formulations on the rheological behavior, photocuring characteristics, printability, and sintering behavior of high-solid-loading photocurable alumina slurries for DLP additive manufacturing. The main findings are summarized as follows:

(1) Among the five monomers examined, 2-HEA and 1,6-HDDA exhibited a favorable combination of low viscosity and high photopolymerization reactivity. The 2-HEA/1,6-HDDA binary monomer system at a weight ratio of 6:4 provided the optimum balance between flowability and photocuring behavior.
(2) The bimodal powder formulation influenced the slurry rheology and photocuring behavior. The 2 μm/100 nm bimodal system exhibited relatively stable rheological behavior over a broad dispersant concentration range, whereas the 4 μm/400 nm system showed higher double-bond conversion during UV curing.
(3) The binary monomer system improved dimensional accuracy during DLP printing by suppressing lateral over-curing compared with the single-monomer system. The finer 2 μm/100 nm system resulted in higher relative density after sintering, owing to its enhanced packing and sintering characteristics.
(4) Among the investigated formulations, the slurry containing the 2 μm/100 nm bimodal powder and the optimized 2-HEA/1,6-HDDA binary monomer system exhibited the best overall performance in terms of printability, dimensional accuracy, densification, and sintering shrinkage.

Overall, within the formulations investigated, the resin formulation showed a greater influence on photocuring behavior and printing accuracy, whereas the bimodal powder formulation, including both particle size combination and coarse-to-fine ratio, showed a greater influence on densification and sintering characteristics.

## Data Availability

The original contributions presented in this study are included in the article. Further inquiries can be directed to the corresponding author.
